# Comparing ChatGPT and Dental Students' Performance in an Introduction to Dental Anatomy Examination: Comment

**DOI:** 10.1055/s-0045-1809979

**Published:** 2025-07-07

**Authors:** Hinpetch Daungsupawong, Viroj Wiwanitkit

**Affiliations:** 1 Private Academic Consultant, Phonhong, Lao People's Democratic Republic; 2 Department of Community Medicine, D. Y. Patil Medical College, Hospital and Research Centre, Dr. D. Y. Patil Vidyapeeth (Deemed to be University), Pune, Maharashtra, India

We would like to comment on “Comparing ChatGPT and Dental Students' Performance in an Introduction to Dental Anatomy Examination: A Cross-Sectional Study.” 1 While the study provided valuable insights, relying solely on frequency and percentage distributions might not fully capture the nuances of the data. The purpose of this article is to compare ChatGPT's knowledge and interpretability to that of undergraduate dentistry students using a multiple-choice dental anatomy test. This study used a cross-sectional analytical study approach, which is ideal for long-term comparisons. However, this research method cannot capture long-term changes or developments in both student and ChatGPT learning. Furthermore, a single average score may not capture other important skills, such as clinical reasoning or knowledge application in real-world scenarios. The researcher analyzed the data using SPSS and Microsoft Excel to determine the percentage and frequency of accurate answers.

However, relying on frequency and percentage distributions may not provide a thorough assessment of ChatGPT's performance. Furthermore, the Shapiro–Wilk test for determining data distribution is only useful when the sample size is quite small. When the test results show a *p* -value of 0.001, it indicates that the data are not normal. As a result, if the data are similar, using the Kolmogorov–Smirnov test may be unnecessary. 2 Reporting more complex statistics, such as assessing significant differences between groups or utilizing Cohen's kappa to assess expert consensus in scoring explanations, would boost the research results' trustworthiness. In artificial intelligence (AI) studies, methods like kappa have been applied to measure the consistency of AI outputs, such as content generation and model evaluation, indicating their potential for increasing reliability in comparative research. 3

In terms of outcomes, the students outperformed ChatGPT, scoring 74.28% on average compared with ChatGPT's 60%. Although ChatGPT was able to answer correctly at a level that meets the minimum criteria, its accuracy and reliability remained low, indicating that AI language models may not be able to analyze data in depth or interpret specific health science contexts as well as humans. Questions to consider in future research include: What are the constraints of ChatGPT in interpreting different types of questions?

To add originality and academic value to future research, researchers should compare ChatGPT with other AI models trained particularly for medicine or dentistry, such as Med-PaLM or BioGPT. 4 A recent study by Wu et al demonstrated that when ChatGPT was integrated with the Knowledge and Few-shot Enhancement In-context Learning framework, its performance improved significantly. 5 ChatGPT-4 achieved the highest score, outperforming the average human score. 5 This highlights the potential of AI models, particularly when tailored for specific domain, to significantly enhance performance in exams and demonstrates the effectiveness of integrating additional frameworks into the evaluation.

Furthermore, developing longitudinal research to track the evolution of ChatGPT's abilities via continuous feedback may reveal its potential as an even more dynamic learning tool. In the future, the integration of ChatGPT into e-learning systems under expert supervision should be investigated to enhance the quality and safety of its application in health education. 6 The integration of AI models into educational settings has already been explored. For example, a recent bibliometric analysis of AI in dental education highlighted a growing interest in applying AI, particularly large language models and chatbots, to transform the field. 7 The study by Iniesta and Pérez-Higueras identified a significant rise in publications on the topic, with key themes like clinical decision support systems and the use of AI in enhancing dental education. 7 The findings emphasize the increasing recognition of AI's potential to improve educational outcomes in health-related fields, further supporting the value of integrating AI models like ChatGPT under expert supervision.
